# Phenotypic Plasticity and Cancer: A System Biology Perspective

**DOI:** 10.3390/jcm13154302

**Published:** 2024-07-23

**Authors:** Ayalur Raghu Subbalakshmi, Sravani Ramisetty, Atish Mohanty, Siddhika Pareek, Dana Do, Sagun Shrestha, Ajaz Khan, Neel Talwar, Tingting Tan, Priya Vishnubhotla, Sharad S. Singhal, Ravi Salgia, Prakash Kulkarni

**Affiliations:** 1 Department of Medical Oncology and Therapeutics Research, City of Hope National Medical Center, Duarte, CA 91010, USA; sayalurraghu@coh.org (A.R.S.);; 2 Department of Medical Oncology and Therapeutics Research, City of Hope Phoenix, Goodyear, AZ 85338, USA; 3 Department of Medical Oncology and Therapeutics Research, City of Hope Chicago, Zion, IL 60099, USA; 4 Department of Medical Oncology and Therapeutics Research, City of Hope San Bernardino Road, Upland, CA 91786, USA; 5 Department of Medical Oncology and Therapeutics Research, City of Hope Avocado Avenue, Newport Beach, CA 92660, USA; 6 Department of Medical Oncology and Therapeutics Research, City of Hope Atlanta, Newnan, GA 30265, USA; 7 Department of Systems Biology, City of Hope National Medical Center, Duarte, CA 91010, USA

**Keywords:** plasticity, epithelial mesenchymal transition, hybrid E/M cells, circulating tumor cells

## Abstract

Epithelial-to-mesenchymal transition (EMT) is a major axis of phenotypic plasticity not only in diseased conditions such as cancer metastasis and fibrosis but also during normal development and wound healing. Yet-another important axis of plasticity with metastatic implications includes the cancer stem cell (CSCs) and non-CSC transitions. However, in both processes, epithelial (E) and mesenchymal (M) phenotypes are not merely binary states. Cancer cells acquire a spectrum of phenotypes with traits, properties, and markers of both E and M phenotypes, giving rise to intermediary hybrid (E/M) phenotypes. E/M cells play an important role in tumor initiation, metastasis, and disease progression in multiple cancers. Furthermore, the hybrid phenotypes also play a major role in causing therapeutic resistance in cancer. Here, we discuss how a systems biology perspective on the problem, which is implicit in the ‘Team Medicine’ approach outlined in the theme of this Special Issue of *The Journal of Clinical Medicine* and includes an interdisciplinary team of experts, is more likely to shed new light on EMT in cancer and help us to identify novel therapeutics and strategies to target phenotypic plasticity in cancer.

## 1. Introduction

Cancer manifests as a condition marked by uncontrolled cell proliferation, often accompanied by its dissemination from the tissue of origin to another distant location through the bloodstream and lymphatic system. The process of establishing neoplastic lesions elsewhere in the body by cancers that originated in different organs, but are no longer in contact with it, is termed metastasis [1], which is known to be the cause of >90% of cancer-related deaths [2]. While metastatic cells frequently spread through the bloodstream, not all circulating tumor cells (CTCs) ultimately cause metastases as the process is dynamic with extremely high attrition rates (>99.5%) [3]. One of the hallmarks of these metastasizing cells is their property of phenotypic plasticity, where they switch phenotypes to adapt to changing environments [4]. This process results in the development of non-genetic diversity by producing a range of phenotypes influenced by different environmental conditions, even though the cell population is isogeneic (shares an identical genetic background) [5,6]. Recent studies have also shown that the process of phenotypic switching involves changes in cellular behavior, biochemistry, and physiology, and it is not just a morphological phenomenon [7].

Epithelial-to-mesenchymal transition (EMT), where epithelial cells switch to a mesenchymal phenotype, and the reverse process, mesenchymal-to-epithelial transition (MET), where mesenchymal cells transition to epithelial cells, is a major axis of phenotypic plasticity. This process is not only observed in pathological conditions such as cancer and fibrosis but is also a major event during development and wound healing [8,9]. During the process of EMT, there is a reduction in traits like apicobasal polarity and cell–cell adhesion, which are hallmark traits of epithelial cells, and a gain of traits associated with migration and invasion, which are behaviors attributed to mesenchymal cells. Initially, the processes of EMT and MET were assumed to be all-or-nothing processes, that is, they were binary states [10], but recent in vitro, in vivo, and in silico studies have established that during these transitions, the cells can exist as a continuum of intermediary states termed as hybrid E/M phenotypes. Furthermore, it has also been observed that the hybrid phenotypes are more adept and aggressive when compared to either the fully epithelial (E) or mesenchymal (M) phenotype [11,12,13]. The process of EMT-MET is only one of the axes of plasticity. The other important axis of plasticity with metastatic implications is the cancer stem cell (CSC) and non-CSC transition [14,15]. When both processes are compared, the hybrid E/M cells have a likeness to the CSCs, whereas the stemness trait seems to be lost as a cell undergoes complete EMT or MET [16,17]. And, because of this, hybrid cells are termed to be ‘fittest’ for metastasis [18].

During the process of metastasis, the carcinoma cells that are epithelial in nature undergo the process of EMT before intravasation. During this transition, the cells acquire a spectrum of intermediary phenotypes where the cells possess the traits, properties, and markers of both epithelial and mesenchymal phenotypes—the intermediary hybrid E/M phenotypes [12]. Hybrid E/M cells have been shown to play a role in tumor initiation, metastasis, and progression in multiple cancers [19,20,21,22,23]. Initially, the hybrid E/M states were assumed to be transient states that cells undergoing EMT or MET attain, but now it has been established that these states are indeed stable [24]. The hybrid cells possess the property of better metastatic potential due to their ability to be more plastic when compared to a completely epithelial or mesenchymal phenotype [21]. Because hybrid E/M cells exhibit characteristics found in both epithelial cells (such as cell–cell adhesion) and mesenchymal cells (like migration), they possess the ability of collective cell migration during developmental processes like tissue and organ formation, as well as during wound healing [25,26]. This property of collective cell migration results in the emergence of clusters of circulating tumor cells (CTCs), which are the primary agents propelling metastasis [27]. The CTC clusters are efficient in intravasation and extravasation [28], and they are anoikis-resistant and possess efficient tumor-forming potential when compared to cells that have undergone a complete EMT due to which they circulate as single cells [29]. Furthermore, clinicopathological data further indicate that the existence of CTCs and hybrid cells results in poor patient outcomes [18,30,31]. Here, we review and discuss the origin and consequences of hybrid E/M cells in cancer progression, metastasis, and drug resistance.

## 2. Hybrid E/M Cells in Cancer Metastasis

**Figure 1:**
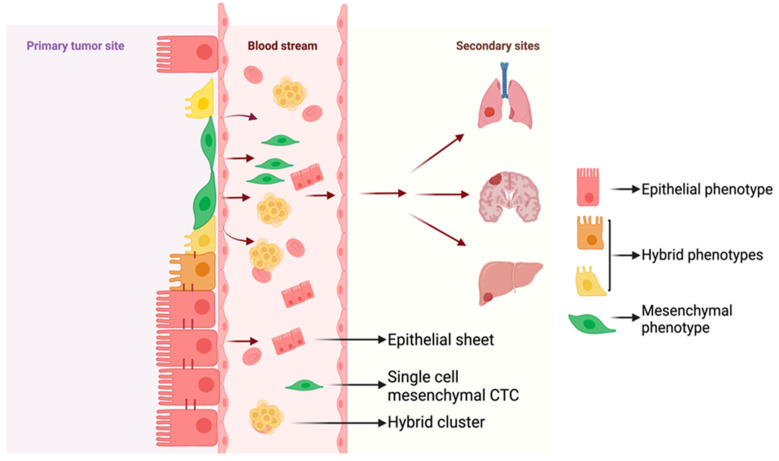
The spectrum of hybrid epithelial/mesenchymal (E/M) phenotypes in circulating tumor cells. During the process of metastasis, cancer cells intravasate into the blood stream and travel to the secondary location. During this process, the cells can travel as epithelial sheets, hybrid clusters, or single (mesenchymal) cells.

The possibility of the existence of a transient/hybrid state during the process of phenotypic switching was reported in a theoretical study by Mahmoudabadi et al. in 2012 [32]. Empirical evidence for the existence of the hybrid E/M phenotype in cancer was first reported by Yu et al. in 2013 [33]. The authors showed that when CTCs derived from breast cancer patients were subjected to RNA–in situ hybridization analysis, they were able to identify cells that were positive for the epithelial (Keratins, EpCAM, and CDH1), as well as mesenchymal, markers (fibronectin 1, CDH2, and SERPINE 1) existing in a spectrum of E/M phenotypes (Figure 1). In the same year, using these molecular markers, Lu and Jolly et al. provided a theoretical framework for the existence of the hybrid E/M phenotype [34]. Subsequently, multiple studies showed that as cells undergo EMT, they exist in a continuum of hybrid E/M phenotypes [30,35,36]. Nonetheless, hybrid cells were assumed to be unstable transitory phases and, hence, their significance in cancer progression remained unappreciated [37]. However, in 2014, experiments on mammary gland development showed that the hybrid E/M phenotype was indeed a stable intermediary phenotype [38] and that these cells could be stably maintained in the hybrid phenotype by precluding their transition to a complete E or M phenotype using phenotypic stability factors like OVOL [38], GRHL2 [24], NFATc [39], and SLUG [40]. In the following sections, we will be discussing the role of hybrid E/M cells in metastasis, stemness, and drug resistance.

### 2.1. Hybrid E/M Cells in Solid Tumors

The National Cancer Institute (NCI) defines the term metastasis as “The spread of cancer cells from the place where they first formed to another part of the body”. However, the process of metastasis is highly inefficient with high attrition rates, as only a tiny fraction of cells (<0.02%) are successful in forming secondary tumors [41]. To overcome attrition and form successful metastases, the cells tend to move as clusters. It has been shown that these clusters of migrating cancer cells comprise hybrid E/M cells, which can adapt to changing environmental conditions due to their virtue of high plasticity, making them the ‘fittest for metastasis’ [18]. It has also been shown that the presence of <2% of hybrid E/M cells results in poor patient prognosis, further supporting the notion of the fitness of the hybrid E/M cells in metastasis [30]. In breast cancer, it has been shown that the emergence of the hybrid E/M state determines tumorigenicity [42]. Here, highly tumorigenic breast cancer cells were isolated using the antigen marker combination of CD104+/CD44hi, and these were found to be stable hybrid cells that co-expressed both epithelial and mesenchymal markers in both in vitro and in vivo settings. It was further observed that when these cells were pushed to undergo a complete EMT in the presence of Zeb1, it resulted in a considerable loss of tumorigenicity. When Brown et al. [36] determined the metastatic potential of breast cancer cells to the lung from across the EMT spectrum, they observed that the cells that resided in the intermediate hybrid E/M phenotype were adept at forming micro- (<10 adjacent cells) and macro (>10 adjacent cells)-metastases when compared to a complete E or M phenotype. They differentiated the various clones based on the CD104+/CD44hi markers into an epithelial (E), three distinct intermediate (EM1, EM2, and EM3), and two mesenchymal (M1 and M2) states. Using SUM149PT cells stably expressing a luciferase–IRES–ZsGreen construct, they observed that the clones from the three intermediary phenotypes formed higher metastatic lesions in the lung when compared to the completely epithelial (E) or mesenchymal (M2) clone. In vivo studies using mammary metaplastic-like and MMTV-PyMT luminal tumors have shown that the metastatic potential of a subpopulation of cells correlated with the hybrid EMT state rather than the expression levels of CD106, a marker associated with the metastatic potential of cancer cells [12]. In breast cancer, it has been further shown that redox signaling by Glutathione Peroxidase 2 (GPx2) controls the EM plasticity, thus affecting the process of metastasis [43]. Using PyMT2 tumors, showing ECAD+/VIM+ hybrid regions with KRT8/KRT14 enrichment, it was shown that GPx2 overexpression promoted MET, resulting in decreased levels of N-CAD, SNAI1, and SLUG with increased expression of ECAD. This resulted in decreased lung colonization upon tail vein injection, further indicating the importance of the hybrid E/M state in breast cancer metastasis.

Like breast cancer, the positive correlation between the hybrid E/M cells and cancer metastasis is also seen in lung cancer progression. This relationship can be seen in the case of H1975 cells, which are stable hybrids, where a reduction in their migratory potential is observed when they undergo a complete EMT [39], highlighting the importance of the hybrid E/M state in the metastasis of lung cancer. A study involving lung cancer revealed that the ability of the hybrid E/M cells to initiate metastasis is dependent on their interaction with immune cells like the natural killer (NK) cells [44]. Here, using lung cancer cell lines (A549 and LT73), which were differentiated as epithelial and hybrid based on levels of EpCAM and SNAI2, it was shown that the hybrid cells, even though they were not very efficient in leaving the primary tumor, showed better metastatic potential when compared to the epithelial cells. This was attributed to their ability to evade immune surveillance by the natural killer cells through reduced secretion of chemokines like CXCL1 and CXCL8. This was also observed in NSCLC patient samples where the presence of the hybrid cells resulted in poor NK cell infiltration and poor patient prognosis.

In hepatocellular carcinoma, the cells were shown to acquire the hybrid phenotype to help them escape anoikis and develop resistance against fluidic shear stress, the metastatic rate-limiting steps [45]. In SNU449 cells, the lncRNA HOTAIR inhibited c-MET-mediated complete EMT and stabilized the cells in the hybrid E/M phenotype. These hybrid E/M cells showed higher metastatic potential in vitro and in vivo in zebrafish. We continue to see these trends in colorectal carcinoma, where hybrid cells result from the knockdown of Neogenin1 (NEO1), a tumor suppressor [46]. Loss of Neo1 resulted in the disruption of the adherens junction and gain of partial EMT status accompanied by increased expression of genes involved in wound healing and migration. In essence, we can assert that inhibiting metastasis can be accomplished by thwarting the development of a hybrid phenotype through factors such as Neo1.

### 2.2. Hybrid E/M Cells in CTCs

Circulating tumor cells, or CTCs, are gateways to glean insights into the metastatic journey, as their numbers show strong correlations with patient outcome and therapy response [47]. There is growing evidence to support the relevance of the hybrid E/M states in CTCs due to their metastatic abilities. Analysis of CTCs derived from patients with advanced-stage disease showed the existence of hybrid E/M cells [48]. Early studies on patient-derived NSCLC and breast cancer CTCs showed that hybrid E/M cells are present in abundance when compared to a completely epithelial or mesenchymal cell [33,49]. CTCs traveling as clusters or collectives are shown to have better metastatic potential (23–50-fold increase) [50]. In breast cancer, it has been shown that the hybrid E/M cells facilitate this collective migration of CTCs [51]. In this study, using the human breast cancer cell lines MCF7 and MDA-MB-231, it has been shown that around 60% of all cells that initiated collective migration were hybrid in nature, and later these hybrid cells evolved as leader cells. This observation also holds true in the case of breast cancer patient-derived CTCs, as a large proportion of them are hybrid E/M cells [52]. When samples from 130 breast cancer patients were analyzed, around 30% of them stained positive for both keratin (epithelial marker) and twist1 (mesenchymal marker) and positively correlated with lung metastasis and decreased progression-free survival. When CTCs from 30 lung cancer patients were analyzed, it was again observed that the number of hybrid CTCs was the highest [53]. Further analysis showed that the number of the hybrid E/M CTCs positively correlated with both lymph node metastasis and distant metastasis, in addition to its positive trends with respect to TNM staging and tumor infiltration depth. To overcome the subjectivity of imaging-based analysis, a TU-chip was developed to conduct objective CTC-based blood tests, where the phenotypes of the CTCs could be differentiated into epithelial, hybrid, and mesenchymal [54]. When blood samples from 46 PDAC patients were analyzed using this technology, it was seen that the hybrid CTCs were a better predictor of metastasis. When hepatocellular carcinoma patient-derived CTCs were segregated into phenotypes based on the EMT spectrum as epithelial, mixed/hybrid, and mesenchymal, it was seen that only 8% of the patients were positive for the hybrid CTCs but were found to be associated with larger tumors and being at stages of B and C (immediate and advanced respectively), according to the Barcelona Clinic Liver Cancer (BCLC) Staging System [55]. Another observation that was made here was that the hybrid CTCs also showed the expression of Nanog, a stem cell marker indicating the stemlike properties of the hybrid cells that make them better suited for metastasis. It was also observed that the number of the hybrid CTCs in conjunction with Nanog expression showed a positive trend with HCC recurrence. Similar to the occurrences in lung and breast cancer, CTC samples derived from head and neck squamous cell carcinoma (HNSCC) patients showed a higher percentage, 28%, of the E^+^M^+^ hybrid CTCs [56]. And, in continuance with the trend, it was also seen that around 63% of patients showing metastatic lesions tested positive for hybrid CTCs.

All these data indicate that irrespective of the cancer type, the presence of the hybrid E/M cells either in solid tumors or CTCs promotes metastasis. Due to their virtue of having properties of both epithelial and mesenchymal cells, they are adept at adapting to changing conditions, which helps them to establish metastatic lesions at distant sites in spite of the high attrition rates involved in the various steps of metastasis. The high plasticity and stem-like properties of these cells further equip them in being successful colonizers at the metastatic site.

### 2.3. Hybrid E/M Cells and Resistance to Cancer Therapy

A major reason for poor cancer prognosis and treatment failures is the ability of the cancer cells to be resistant to therapeutic strategies [57]. This resistance can be either intrinsic (also called innate) or acquired. Inherent resistance, sometimes referred to as primary resistance, is governed by the internal factors (mutations) within tumor cells or tissues existing prior to therapeutic interventions. These factors confer survival benefits to cancer cells and enable them to adapt to initial therapeutic pressures [58,59]. In contrast, acquired drug resistance emerges following cancer treatment, typically driven by adaptive changes in initially responsive tumors against the prescribed therapy, leading to diminished treatment efficacy [60,61]. Now, there is a large body of growing evidence to show that the process of EMT has a major role to play in the therapeutic resistance of cancer cells [62,63,64]. Now, investigators have delved deeper into the process of EMT to determine the effects of the different EMT states on therapy resistance, especially the hybrid E/M phenotype.

In MDA-MB-231 cells, low levels of miR18a, a miRNA that plays key roles in tumor malignancies, is attributed to the enrichment of luminal–basal hybrid cells [65]. The reduction in the expression levels of miR18a was seen to result in the increased expression of the ABC family genes, which further showed an upregulation of drug response pathways. There was also an uptick in the levels of integrins, which are known contributors to drug resistance in breast cancer [66]. Further, paclitaxel treatment of MDA-MB-231 cells with low miR18a expression showed better cell viability, indicating that the hybrid E/M cells play a role in drug resistance. In breast cancer, CD24-/low/CD44+ and ALDH+ phenotypes are termed to be cancer stem cells [67]. Decreased CD24 expression in breast cancer cells correlates with resistance to radiation and the management of oxidative stress, in addition to acquiring a hybrid E/M phenotype [68]. A study on chemoresistance in breast cancer by Lüönd et al. revealed that when compared to a completely epithelial cell, the hybrid and mesenchymal cells were both resistant to therapy [69]. To determine the association between cell phenotypes across the EMT spectrum, chemoresistance breast cancer cells were treated with three therapeutic drugs, namely paclitaxel (PAX), cyclophosphamide (CPH), or doxorubicin (DOX). The relationship between the hybrid E/M cells and therapy resistance in breast cancer has also been shown using a mechanistic framework [22]. In ER+ breast cancer, resistance to drugs like tamoxifen is regulated by the EMT-associated factors ZEB1, miR-200, and SLUG, as they interact with the estrogen receptor alpha (ERα). As the cells transition to the hybrid or mesenchymal phenotype, they gain resistance against anti-estrogen therapy as there is a reduction in the levels of Erα due to its nature of interaction with the EMT factors.

Understanding the relationship between hybrid E/M cells and therapy resistance in cancer is crucial for improving treatment strategies and patient outcomes. Despite the growing recognition of hybrid E/M cells as key players in cancer progression and metastasis, the literature on their role in therapy resistance remains relatively scarce. This gap in knowledge underscores the urgent need for comprehensive studies to unravel the mechanisms underlying therapy resistance mediated by hybrid E/M cells.

## 3. Conclusions and Future Directions

The intricacy of cancer encompasses genetics, cellular and tissue biology, pathology, and responses to treatment, making it daunting. The process of metastasis is one of the hallmarks of cancer [70,71]. The emergence of metastasis leads to grim patient prognosis, as it allows cancer cells to acquire therapeutic resistance. This may explain why >90% of cancer-related deaths are linked to metastasis [24,71,72]. The process of EMT contributes to metastasis by enhancing the mobility and invasive capacities of cancer cells, as well as making them resistant to anoikis. This program, which is evolutionarily conserved and activated during development and wound healing, plays a significant role in metastatic progression [73]. Initially assumed to be binary, the process of EMT is now recognized to encompass multiple stable intermediary phenotypes, known as hybrid E/M phenotypes [35,74]. In the field of molecular oncology, the partial EMT, or hybrid E/M phenotype, is gaining attention due to its recognized role in promoting stemness, conferring therapy resistance and immunosuppression, and enhancing metastasis, which collectively contribute to patient mortality [11,17,20,21,22]. Recent extensive studies, spanning the transcriptomic and proteomic spectrum, have been motivated by the clinical implications of hybrid E/M cells. These investigations aim to understand their biophysical and biochemical traits across multiple cancers [75,76,77,78,79,80,81].

A question that now arises is as follows: if hybrid phenotypes are advantageous since they appear more adept, are they also observed in lower organisms? Indeed, in the case of the yeast *Candida albicans*, a commensal fungus and opportunistic pathogen, an intermediary phenotype called the gray phenotype was identified by Tao et al. [82]. Initially, yeast were assumed to be present in two phenotypic states, namely white and opaque. In the white state, *Candida albicans* displays a yeast-like morphology and is often associated with bloodstream dissemination and systemic infections [83]. Conversely, the opaque phenotype is characterized by elongated cells with a wrinkled surface, and it is predominantly found in mucosal infections and host niches [84]. The transition between these two phenotypic states is regulated by a master transcriptional regulator, Wor1, along with other regulators such as Efg1 and Czf1 [84]. Studies have shown that the white-to-opaque transition is essential for host adaptation and immune evasion, while the opaque-to-white transition is crucial for systemic dissemination and colonization of new host niches [85]. Now, the newly discovered grey phenotype represents a transitional state characterized by elongated, filamentous cells with enhanced adherence properties and increased resistance to environmental stressors. This phenotypic transition is regulated by a complex network of transcription factors, including Wor1, Wor2, and Efg1, which orchestrate the expression of genes involved in filamentation, adhesion, and virulence [82,86].

When we move further down the evolutionary tree, we observe that the property of phenotypic switching is also present in bacteria, as bacterial cells can exist in multiple stable phenotypic states within the same population. This phenotypic heterogeneity arises from complex regulatory networks and environmental cues, allowing bacteria to adapt to changing conditions and stressors. One well-studied example of multistable behavior in bacteria is the phenomenon of persister cells, which represent a subpopulation of dormant cells that are tolerant to antibiotics and other stressors [87]. Additionally, bistable expression of virulence factors in pathogens such as *Escherichia coli* and *Salmonella enterica* contributes to multistable behavior, allowing bacteria to switch between distinct phenotypic states in response to environmental cues [88,89]. Understanding the molecular mechanisms underlying multistable bacterial populations is crucial for unraveling bacterial adaptation strategies, pathogenesis, and antibiotic resistance mechanisms, ultimately informing the development of novel therapeutic approaches.

**Figure 2:**
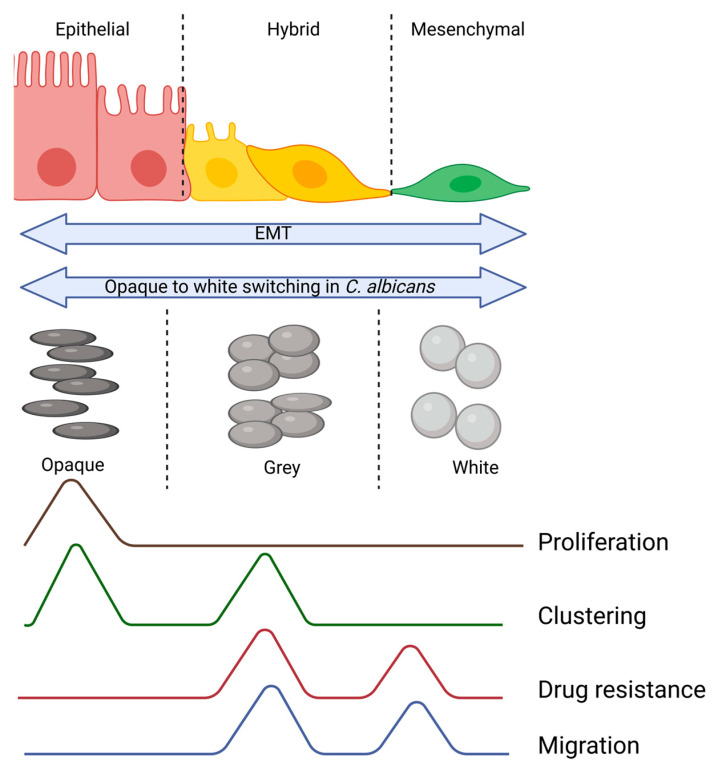
Similarities in characteristics between cancer cells undergoing EMT and *C. albicans*. The intermediary phenotypes, irrespective of the evolutionary scale, possess properties of both the extreme phenotypes, thus providing them with a survival advantage.

Drawing parallels between hybrid E/M cells in cancer, the gray phenotype of *Candida albicans*, and intermediary phenotypes in bacteria reveals intriguing similarities in phenotypic plasticity across diverse biological systems. In cancer, hybrid E/M cells exhibit a mixed epithelial and mesenchymal phenotype, allowing them to adapt to dynamic microenvironments, promote metastasis, and confer therapy resistance. Similarly, the gray phenotype of *Candida albicans* represents a transitional state characterized by enhanced adaptability and virulence, enabling the fungus to thrive in diverse host environments and evade immune responses. Likewise, intermediary phenotypes in bacteria showcase phenotypic heterogeneity within bacterial populations, facilitating adaptation to changing conditions, antibiotic tolerance, and pathogenicity (Figure 2).

The foregoing also results in an important question: is the emergence of the hybrid epithelial/mesenchymal (E/M) state in cancer a product of evolutionary memory? This question probes the possibility that the transition to a hybrid E/M phenotype, characterized by enhanced mobility, invasiveness, and resistance to anoikis, might be rooted in evolutionary adaptations. Evolutionary memory suggests that biological systems retain ancestral traits that have conferred survival advantages over millennia. Considering the dynamic interplay between cancer cells and their microenvironments, it is conceivable that the hybrid E/M state could be a manifestation of ancient survival mechanisms repurposed by cancer cells. By tapping into evolutionary memory, cancer cells may gain an evolutionary edge, enabling them to navigate and thrive in the challenging landscape of the tumor microenvironment.

Thus, it follows that a systems biology perspective to the problem conceived in the ‘Team Medicine’ approach—the theme of this Special Issue of *The Journal of Clinical Medicine*—that includes an interdisciplinary team of experts is more likely to shed new light on EMT in cancer. Exploring the concept of evolutionary memory in the context of cancer biology could unveil novel insights into the origins and adaptive strategies of cancer cells, potentially paving the way for innovative therapeutic interventions targeting these evolutionary pathways.
